# Stranger to Familiar: Wild Strepsirhines Manage Xenophobia by Playing

**DOI:** 10.1371/journal.pone.0013218

**Published:** 2010-10-07

**Authors:** Daniela Antonacci, Ivan Norscia, Elisabetta Palagi

**Affiliations:** 1 Centro Interdipartimentale Museo di Storia Naturale e del Territorio, Università di Pisa, Pisa, Italy; 2 Unit of Cognitive Primatology and Primate Center, Institute of Cognitive Sciences and Technologies, The National Research Council (CNR), Rome, Italy; University of Lethbridge, Canada

## Abstract

The power of play in limiting xenophobia is a well-known phenomenon in humans. Yet, the evidence in social animals remains meager. Here, we aim to determine whether play promotes social tolerance toward strangers in one of the most basal group of primates, the strepsirhines. We observed two groups of wild lemurs (*Propithecus verreauxi*, Verreaux's sifaka) during the mating season. Data were also collected on nine visiting, outgroup males. We compared the distribution of play, grooming, and aggressive interactions across three conditions: OUT (resident/outgroup interactions), IN (resident/resident interactions in presence of outgroups) and BL-IN (baseline of resident/resident interactions in absence of outgroups). Play frequency between males was higher in OUT than in IN and BL-IN conditions; whereas, grooming was more frequent in IN than in OUT and BL-IN conditions. Aggression rates between resident and outgroup males were significantly higher than those between residents. However, aggressions between resident and outgroup males significantly decreased after the first play session and became comparable with resident-resident aggression levels. The presence of strangers in a well-established group implies the onset of novel social circumstances, which sifaka males cope with by two different tactics: grooming with ingroup males and playing with outgroup ones. The grooming peak, concurrently with the visit of outgroups, probably represents a social shield adopted by resident males to make their pre-existing affiliation more evident to the stranger “audience”. Being mostly restricted to unfamiliar males, adult play in sifaka appears to have a role in managing new social situations more than in maintaining old relationships. In particular, our results indicate not only that play is the interface between strangers but also that it has a specific function in reducing xenophobia. In conclusion, play appears to be an ice-breaker mechanism in the critical process that “upgrades” an individual from stranger to familiar.

## Introduction

*You can discover more about a person in an hour of play than in a year of conversation*

Plato, The Republic

Xenophobia (from Greek: *xénos*, foreign and *phobos*, fear) literally indicates aversion to strangers and in its extreme form is expressed through a cooperative violent reaction of the residents toward strangers. This phenomenon, addressed as “xenophobia principle” by socio-biologists, is widespread in animals ([1], p. 286). In primates, xenophobic reactions include agonistic chasing (in sifaka: *Propithecus verreauxi*;[2]), target aggressions (in ring-tailed lemurs: *Lemur catta*; [3]), cooperative attacks (in rhesus monkeys: *Macaca mulatta*; [4]), coalitionary killing (in chimpanzees, *Pan troglodytes*; [5]) and warfare (humans, *Homo sapiens*; [6]).

The power of play in limiting xenophobia by promoting tolerance in humans was known since ancient times. According to Greek mythology, Apollo, queried through Delphi's oracle, told Ifitos (the King of Elis) that the wars devastating Peloponnese would be ended by staging a sport competition at Olympus. After the Olympic Games were re-established (and this is where the myth ends and history begins), the longest-standing peace accord in history (the Olympic Truce) was signed between the regions of Peloponnese [7]. The role of human play in limiting xenophobic aggressions is more than anecdotal and does not apply only to structured play. This is not surprising, considering that complex social play represents one step of play ontogeny, which begins with spontaneous play fighting and reaches its climax with the production of sophisticated games [8]. In children, play fighting (or rough and tumble) leads to the direct inhibition and regulation of aggression, thus improving social integration [9]. Hunter-gatherer societies where play (both with rules and without) is used in social practices (religion, bargaining, children's education, etc.), show a more fluid, democratic structure and are more open to new incomers [10].

Animals, as well as children, do follow rules during social play but such rules are flexible and negotiated by players *hic et nunc* (“here and now”) [11]. Flexibility and improvisation that characterize social play are considered to be the locomotive of cognitive and behavioral innovation [12].

In juveniles, play can have long term positive effects by improving motor and psychosocial skills [13]–[16]. Among adults, play appears to be especially fruitful at a short term level for manipulating specific social situations (tension reduction, cohesiveness increase, low risk relationship assessment) [17]–[22].

Thus, what is really important in adult-adult play is animals' ability to opportunistically use play in the most appropriate way. Consequently, play effectiveness does not lie on quantity (how much adults play) but on quality that is how (social context), with whom (play-mate choice), and when (timing) adults play.

While juvenile play is ubiquitous among primates, adult play is less frequent and is scarcely documented in quantitative terms [18], [21], [23], [24]. Adult play is unrelated to phylogenetic relationships among species (showing a patchwork distribution among primate taxa) [25], and strongly affected by social organization and inter-individual bonding quality [20]. Focusing on adults may be a first step toward a deeper understanding of the short term benefits of play [26].

Adult play benefits seem to be *maxima* in case of uncertainty in social relationships among individuals [26], such as when mating involves unfamiliar subjects [27]–[29] and/or when group composition is fluid, with some group members meeting each other occasionally (e.g. in fission-fusion societies: *Homo sapiens*, [10]; *Pan* spp., [30], [31]; *Ateles* sp., [32]; *Cacajao* sp., [33]). During ephemeral and sporadic associations, animals have to engage in behavioral interactions to establish or re-establish a sufficient level of familiarity [34].

Here, we aim to determine whether play is used to manage xenophobia in the most basal group of primates, the strepsirhines. To test this hypothesis, we selected the sifaka, *Propithecus verreauxi*, a species where adult-adult play occurs. Most strepsirhines are characterized by either dispersed sociality (solitary or pair-living individuals) [35] or social xenophobic groups completely sealed to outsiders (e.g. *Lemur catta*, [3]). Sifaka live in cohesive multi-male/multi-female groups and show temporary variations in group composition, especially during the mating season [36]–[39]. In this period, males can start roaming and visiting other groups in search of receptive females, which experience a single estrus period per year (up to 72 h). Subjects of both sexes can mate with multiple partners in their own and neighboring groups [40], [41]. Mate choice is a prerogative of females, due to their dominance over males [39], [40].

The plasticity characterizing sifaka groups provides a rare opportunity to determine if adult play facilitates the integration of unfamiliar individuals. We tested the following predictions.

### Prediction 1 – *Can play be considered as a purely affinitive behavior?*

Grooming is used as the main social cement within primate social groups and it is typically, mostly exchanged between individuals sharing good relationships [42]. Different authors have shown that social play, as well as grooming, can work to maintain relationships between subjects with pre-existing social bonds [24], [30]. On the other hand social play, can also involve unfamiliar individuals, and we therefore hypothesize that play is not solely affinitive. If so, we expect grooming and play not to follow the same distribution patterns, especially when unfamiliar subjects are involved (Prediction 1).

### Prediction 2 – *Play for courtship*

When a male meets an unfamiliar female immediately before it is receptive (courtship context), play appears to reduce aggression, thus establishing familiarity suitable for more relaxed and successful copulations [27]–[29].

If social play is also used by outgroup, sifaka males for courtship, we predict i) higher levels of play between resident females and outgroup males compared to resident females and males; and ii) higher motivation to engage in social play by outgroup males than resident females

### Prediction 3 – *Play for promoting tolerance and limiting xenophobia*

In *Propithecus verreauxi* intergroup encounters are common at feeding sites within overlapping home-ranges [2],[43]. Moreover, resident males have been observed to sometimes form coalitions to keep extra-group males out and to prevent them from mating with resident females [44]. However, residents also exhibit behaviors that appear to facilitate group membership for strangers [45], possibly due to the potential benefits provided by extra males in groups of sifakas such as increased vigilance and resource defense [46]. Hence, under certain circumstances males need to modulate and moderate their xenophobic response, in order to mediate between acceptance and rejection of outgroup males trying to break into the group.

In this case of extreme social uncertainty, play may be used as an ice-breaking mechanism to promote tolerance and limit xenophobia. If so, we expect i) higher levels of play between ingroup and outgroup males (more “unfamiliar” to each other) than between ingroup males; ii) comparable levels of play initiation between outgroup and ingroup males; iii) a decrease of agonistic interactions after play between ingroup and outgroup members.

### Prediction 4 – *Does familiarity affect play intensity?*

Animals can fine-tune play sessions, in terms of intensity, according to play mate, context (more or less risky), and timing [47]. Play can be graded along a gradient of intensity, going from gentle play, involving no body contact (e.g. play run) or a sequence of contact and/or no-contact patterns, to rough play (or rough and tumble), involving fighting with a series of body contact patterns normally performed in rapid succession (e.g. biting, pushing, pulling, rolling, falling on the ground) [26], [48]. In its roughest version, play is one of the most sophisticated forms of social interaction, during which playmates have to trust each other to maintain play rules and avoid escalation into serious fights [49]. If, in the study species, social play also implies trust between individuals rough play should be more common among ingroup males than between outgroup and ingroup males.

## Results

### Play and grooming distribution

We compared play and grooming levels across three conditions: male-OUTmale (interactions between males of the observed groups and outgroup males), male-INmale (interactions between males of the observed groups during the visit of outgroup males), and male-BL-INmale (control variable including the interactions between males of the observed groups recorded in absence of outgroup males).

**Figure 1:**
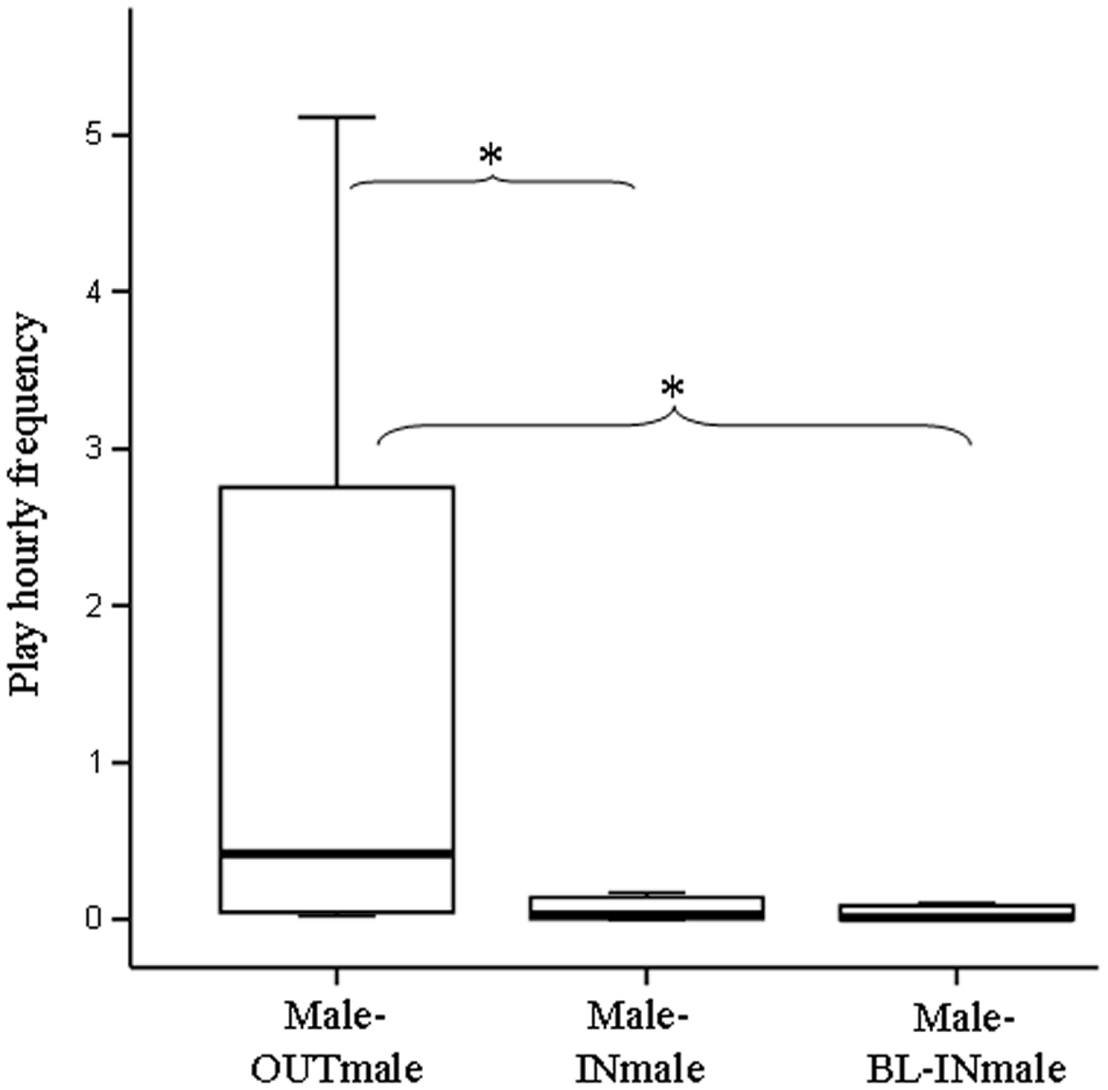
Levels of play between resident males and between resident and stranger males. According to the three conditions, the hourly distribution of play frequency are reported as follows: male-OUTmale (play interactions between males of the observed groups and outgroup males), male-INmale (play interactions between males of the observed groups during the visit of outgroup males), and male-BL-INmale (play interactions between males of the observed groups recorded in absence of outgroup males). Solid horizontal lines indicate medians; length of the boxes corresponds to inter-quartile range; thin horizontal lines indicate range of observed values. Only statistically significant values are reported on the figure. The single asterisk (*) indicates p<0.05.

**Figure 2:**
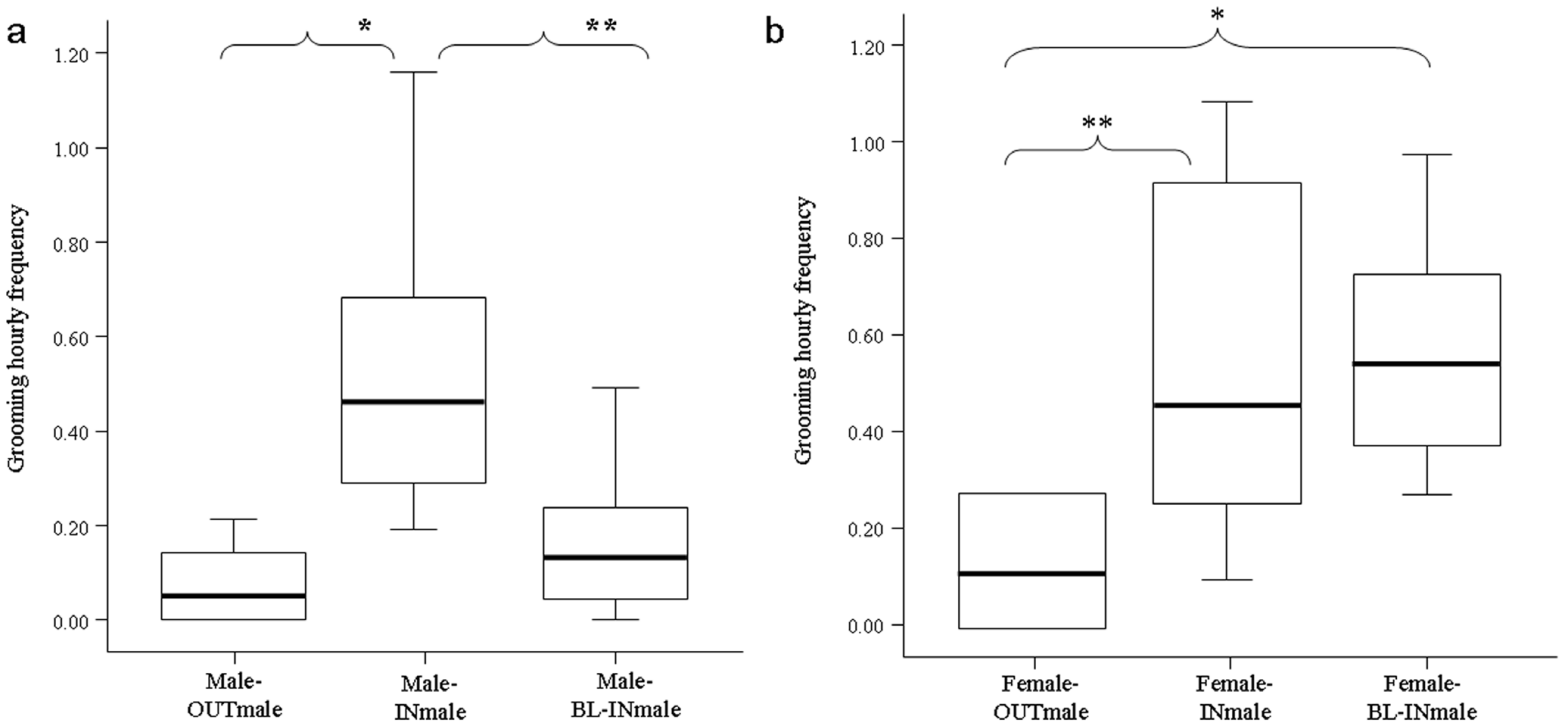
Levels of grooming between resident animals and between resident and stranger animals. According to the three conditions, the hourly distribution of grooming frequency is reported as follows: male-OUTmale (grooming interactions between males of the observed groups and outgroup males), male-INmale (grooming interactions between males of the observed groups during the visit of outgroup males), and male-BL-INmale (grooming interactions between males of the observed groups recorded in absence of outgroup males) (**a**). According to the three conditions, the hourly distribution of grooming frequency is reported as follows: female-OUTmale (grooming interactions between females and outgroup males), female-INmale (grooming interactions between females and ingroup males during the visit of outgroups), female-BL-INmale (grooming interactions between females and ingroup males recorded when the outgroup males were absent) (**b**). Solid horizontal lines indicate medians; length of the boxes corresponds to inter-quartile range; thin horizontal lines indicate range of observed values. Only statistically significant values are reported on the figure. Single asterisk (*): p<0.05; Double asterisk (**): p<0.01.

We found a significant difference in the play distribution across the three conditions: male-OUTmale, male-INmale, male-BL-INmale (Friedman's *χ* ^2^ = 13.034; df = 2; N_males_ = 8; p = 0.001). Dunnett's test revealed a significant difference between male-OUTmale *vs* male-INmale (OUTmale > INmale: q = 2.01; p<0.05) and male-OUTmale *vs* male-BL-INmale (male-OUTmale *>* male*-*BL-INmale q = 1.97; p<0.05); conversely, no difference was found between male-INmale *vs* male-BL-INmale (q = 1.03; p>0.05) (Fig. 1). Outgroup and ingroup males initiated play sessions at similar rates between each other (Wilcoxon's T = 8.50; ties = 3; N_males_ = 8; p = 0.75). Grooming distribution significantly differed according to male-OUTmale, male-INmale, male-BL-INmale conditions (Friedman's *χ* ^2^ = 12.97; df = 2; N_males_ = 8; p = 0.0001). Specifically, Dunnett's test revealed a significant difference between male-OUTmale *vs* male-INmale (male-INmale > male-OUTmale: q = 1.98; p<0.05) and male-INmale *vs* male-BL-INmale (male-INmale > male-BL-INmale: q = 2.41; p<0.01); no difference was found between male-OUTmale *vs* male-BL-INmale (q = 0.70; p>0.05) (Fig. 2a).

We compared play and grooming sessions between females and outgroup males (female-OUTmale), females and ingroup males (during the visit of outgroups, female-INmale), females and ingroup males (recorded when the outgroup males were absent, female-BL-INmale). We found no significant difference in female play distribution across the three conditions: female-OUTmale, female-INmale, and female-BL-INmale (Friedman's *χ* ^2^ = 1.45; df = 2; N_females_ = 6; p = 0.51). Outgroup males and resident females initiated play sessions at comparable levels between each other (Wilcoxon's T = 0; ties = 3; N_females_ = 6; p = 0.250). Grooming distribution significantly differed according to the three conditions (female-OUTmale, female-INmale, female-BL-INmale) (Friedman's chi-square = 7; df = 2; N_females_ = 6; p = 0.029). In particular, Dunnett's test showed a significant difference between female-OUTmale *vs* female-INmale (female-OUTmale *<* female-INmale: q = 2.12, p<0.01) and female-OUTmale *vs* female-BL-INmale (female-OUTmale < female-BL-INmale: q = 1.98, p<0.05); no difference was found between female-INmale vs female-BL-INmale (q = 1.06; p>0.05) (Fig. 2b).

### Aggressions and play

**Figure 3:**
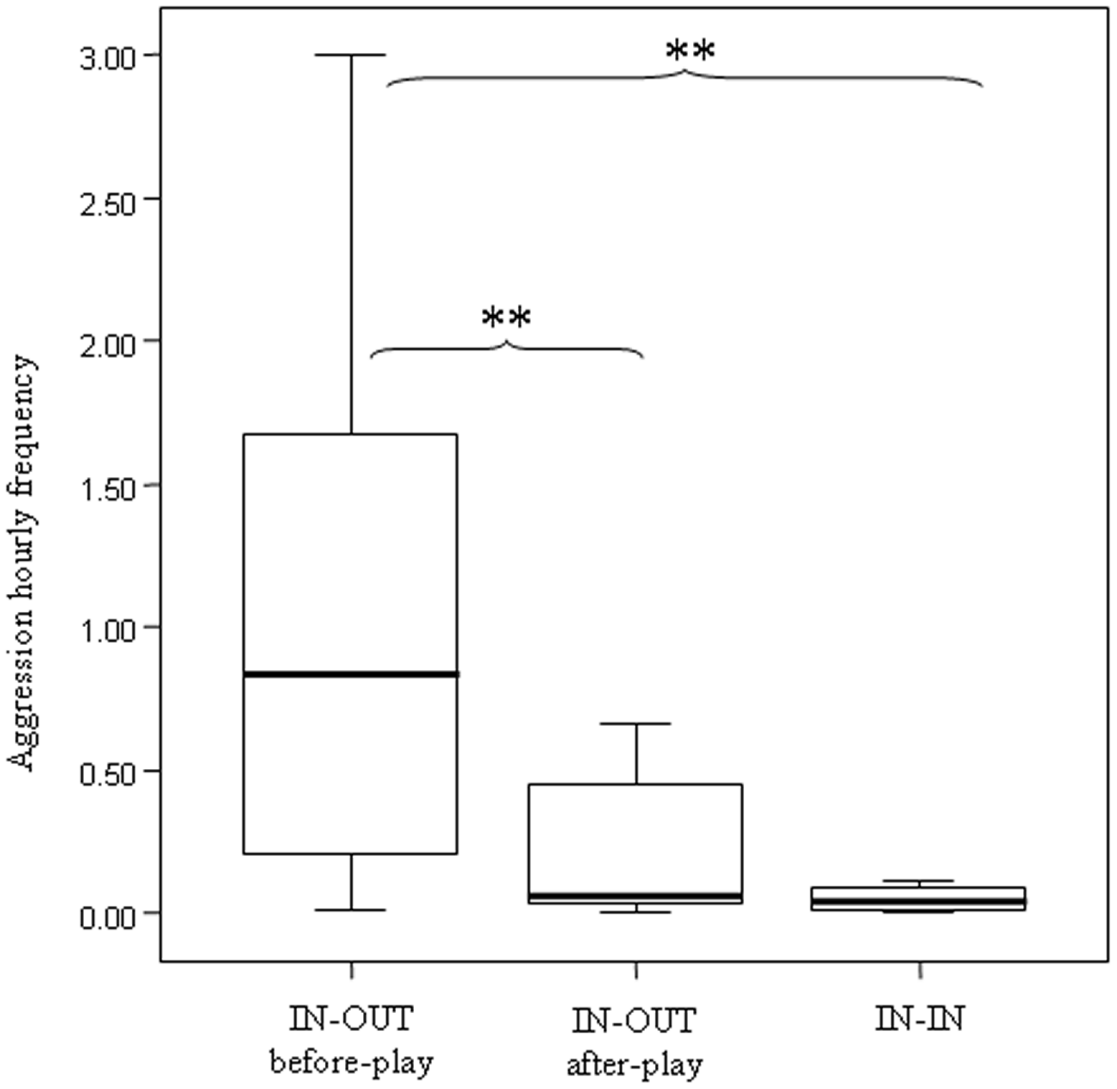
Levels of aggressions between resident and stranger males before and after play. According to the three conditions, the hourly distribution of aggression frequency is reported as follows: IN-OUTbefore-play (aggressions between resident and outgroup males before play), IN-OUTafter-play (aggressions between resident and outgroup males following play), and IN-IN (aggressions between residents). Solid horizontal lines indicate medians; length of the boxes corresponds to inter-quartile range; thin horizontal lines indicate range of observed values. Only statistically significant values are reported on the figure. Single asterisk (*): p<0.05; Double asterisk (**): p<0.01.

We compared aggression rates across three conditions: IN-OUTbefore-play (aggressions between resident and outgroup males before the first session of play), IN-OUTafter-play (aggressions between resident and outgroup males following the first session of play), and IN-IN (control variable including the aggressions between residents). Aggression rates significantly differed across the three conditions (Friedman's *χ* ^2^ = 8.194; df = 2; N_males_ = 8; p = 0.014). Before play, aggression rates between resident and outgroup males were significantly higher than aggression rates between residents (IN-OUTbefore-play>IN-IN; Dunnett's test, q = 3.54; p<0.01) but such difference vanished after play (IN-OUTafter-play≈IN-IN; Dunnett's test, q = 0.34; p>0.05). Moreover, aggression rates between resident and outgroup males significantly decreased after play (IN-OUTbefore-play>IN-OUTafter-play; Dunnett's test, q = 5.79; p<0.01) (Fig. 3).

We never observed any aggressive event directed by females towards outgroup males.

### Familiarity and play intensity

We distinguished play sessions as a function of their intensity: rough (R_play_) if the session included at least one Rough-and-Tumble pattern (as defined in Table 1; e.g. Video S1 and Video S2) and gentle (G_play_) if not (e.g. Video S3).

**Table 1:** Play behavioural patterns observed in adult sifaka over 481 play sessions recorded.

PLAY ITEMS	DESCRIPTION
**ACROBATIC PLAY (acp)**	One (solitary play) or more individuals (social play) climb, jump and dangle from supports of the environment (i.e. branches)
**GRAB GENTLE (grg)**	An individual gently massages the playmate
**PLAY BITE (pbit)**	An individual bites a part of the playmate's body
**JUMP ON ANOTHER (pja)**	An individual jumps with its four limbs on a playmate
**PLAY PULL (ppl)**	An individual grasps another playmate
**PLAY PUSH (pps)**	An individual pushes another playmate with its hands or feet
**PLAY SLAP (psl)**	An individual slaps any part of the fellow's body
**PLAY BITE GENITALS (pbitg)**	An individual gently bites the playmate's genitals
**PLAY RETRIEVE (pre)**	An individual holds the playmate in order to prevent him from leaving the play session
**ROUGH AND TUMBLE (rt)**	Vigorous wrestling, involving patterns such as rolling, pulling, pushing, slapping, and falling on the playmate.
**GENTLE WRESTLING (gw)**	Limbs entwined while sitting or laying individuals roll together placing their open mouths on each other.

**Figure 4:**
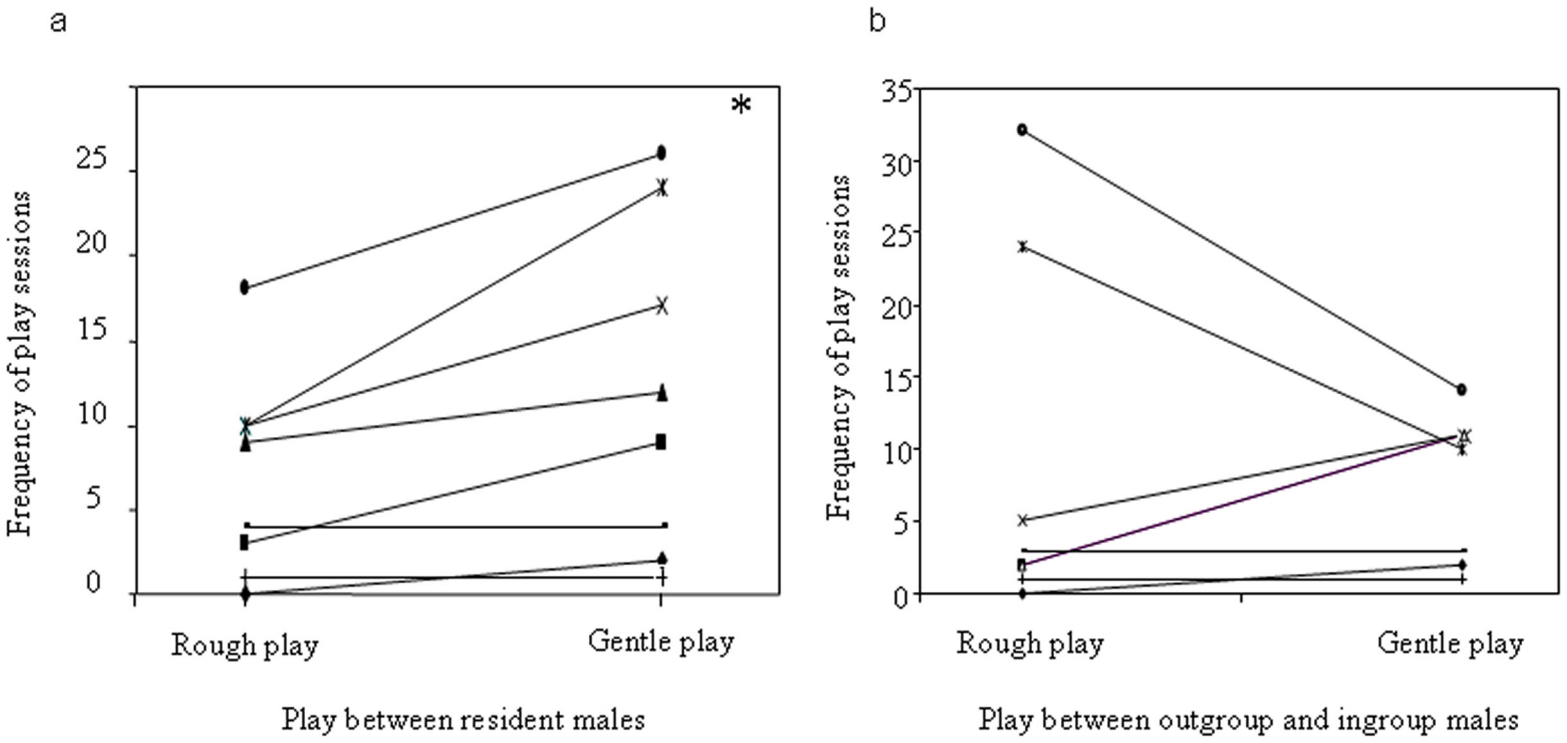
Differences in play modality. Rough and Gentle play interactions that occurred between resident males (ingroup-ingroup males) (**a**) and between resident and stranger males (outgroup-ingroup males) (**b**). The single asterisk (*) indicates p<0.05.

G_play_ was significantly more frequent than R_play_ when play sessions involved resident males (Wilcoxon's T = 0, ties = 2; N_males_ = 8, p = 0.031) (Fig. 4a); on the other hand, R_play_ and G_play_ did not differ during the play sessions between resident and outgroup males (Wilcoxon's T = 11, ties = 2; N_males_ = 8, p = 0.938) (Fig. 4b). Consistently, the mean percentage of R_play_ frequencies was 28.54% ±SE 6.95% between resident males and 48.19% ±SE 13.12% between resident and outgroup males; whereas, the mean percentage of G_play_ frequencies was 71.46% ±SE 6.95% between resident males and 51.81% ±SE 13.12% between resident and outgroup males.

The duration of R_play_ sessions observed between ingroup males (median = 17.50, lower quartile = 3, upper quartile = 50.62) was longer than that observed between ingroup and outgroup males (median = 10.00, lower quartile = 1.25, upper quartile = 10.00) (Wilcoxon's T = 0, ties = 2, N_males_ = 8, p = 0.03). On the contrary, there was no difference in the duration of the G_play_ sessions (G_play_ between resident males: median = 7.50, lower quartile = 1.25, upper quartile = 10.00; G_play_ between resident and outgroup males: median = 5.00, lower quartile = 1.25, upper quartile = 10.00) (Wilcoxon's T = 2, ties = 4, N_males_ = 8, p = 0.50).

## Discussion

The presence of unfamiliar individuals in a well-established group implies the onset of novel social circumstances, which residents have to cope with [34], [50].

This report is the first quantitative study showing that adult play can be used as the main tool for increasing tolerance and reducing xenophobic expressions between stranger animals.

Our data on wild sifaka suggest a functional dichotomy between grooming and social play (Prediction 1 supported). In fact, for both males and females play and grooming distributions do not proceed in tandem. Grooming is mostly exchanged between residents, thus being confirmed as an affinitive behavior used to maintain pre-existing social relations. The presence of outgroup individuals induces an increase of grooming between resident males (Fig. 2a). Such increase could be read as a sort of social shield adopted by resident males to consolidate and/or make their bonding more evident to the stranger “audience”. This response is predicted by the xenophobia principle, which suggests that a peak of cooperative behaviors among insiders is evoked by newcomers [1]. The presence of outgroup do not seem to influence grooming distribution between resident males and females (Fig. 2b), probably because females are relieved from vigilance and resource defense, which are mostly up to males [46]. Additionally, the presence of outgroup males is an added positive value for females because it is associated to increased mate choice opportunities [39].

Whilst ingroup males engaged in play with outgroup males more than with ingroup ones (Fig. 1), females engaged in play with ingroup and outgroup males at comparable levels. Hence, male-male adult play seems not to be a purely affinitive behavior but mostly a means to test emergent relationships between strangers (Prediction 1 supported). Female-male adult play seems not to have a similar function, with outgroup males not using play to access females for courtship (Prediction 2 not supported). Female criteria for partner selection can explain such a result. To be selected by females, males have to be good scent releasers and groomers [39] more than good players. In fact, females grant mating priority to those males that are most active in scent-marking and a greater amount of renewed copulations to those males they receive most grooming from. In this respect, sifaka would differ from other primate species that seem to use courtship-play as a social tool for overcoming female reticence when male-female association is low (*Galago demidovii*, *Perodicticus potto*, [51]; *Mirza coquereli*, [52]; *Ateles* sp., [53]; *Pongo pygmaeus*, [54]. However, the complete lack of quantitative studies (other than the present one) on this issue leaves the role of primate play in courtship largely unexplored.

Being mostly restricted to unfamiliar males, adult play in sifaka appears to have a role in managing new social situations more than in maintaining “old” relationships. In particular, our results indicate not only that play is the interface between strangers but also that it has a specific function in reducing xenophobia (Prediction 3 supported), normally expressed by this species via aggressive chases. Aggressions by ingroup males were preferentially directed toward outgroup males more than toward other group members. After play, conflicts between unfamiliar males plunged to the levels observed between familiar males (Fig. 3). Ingroup males initiated play sessions as much as outgroup males, thus indicating that ice-breaking via play is worthwhile and beneficial for both parties. In primates, the presence of unfamiliar individuals in the group can provoke social tension and stress in animals [55]. Recent studies on rodents and primates demonstrated a link between mild stress and social play [26], [56]. For example, in rats a short period of social isolation is an effective way to increase the amount of social play when the temporary-isolated subjects are placed back with partners. In addition, experimental studies revealed that rats treated with ACTH (Adreno Cortico Tropic Hormone, a stress-related hormone) increased their play levels compared to those of saline-treated controls, thus suggesting that moderate amount of stress or anxiety promotes social play [26]. Accordingly, in order to cope with the forthcoming anxiety associated with the presence of food, captive primates increase their play levels during the time-period preceding food distribution. Moreover, dyads playing during the pre-feeding time show high levels of tolerance around food [17], [47], [57]. By helping animals to overcome stress and dissipate tension, social play in sifaka appears to represent a strategic toolkit for aggression control. This strategy is clearly advantageous because it promotes good relations between unfamiliar individuals thus reducing at minimum the costs that xenophobia would bring, in terms of aggression and group stability. This behavior has therefore immediate benefits to the animals but also long term advantages suggesting the presence in this species of cognitive capacities for anticipating future events.

We found that adult males adjust their playful tactics as a function of playmates' group membership. Since social play implies trust between individuals rough play should be more common among ingroup males than between outgroup and ingroup males (Prediction 4). Contrary to the expectations, resident males engaged mainly in gentle play sessions when playing together (Fig. 4a), whereas rough and gentle play frequencies did not differ when the play sessions involved resident and outgroup males (Fig. 4b). However, rough play sessions were longer when ingroup members only were involved (Prediction 4 partially supported). As a whole, resident males do not limit the use of the rougher mode of play when interacting with unfamiliar males, but they do limit the duration of such sessions.

Rough play is one of the most complex interactions used by animals to gather information on the potential of co-specifics as competitors or social partners [26]. In sifaka, rough play might be a sort of competitive/cooperative interaction that serves to test a partner's willingness to invest in a new relationship, and simultaneously to demonstrate one's own willingness to accept vulnerability. In short, rough play is a declaration of acceptance of the new social situation.

Rough play can be particularly risky in species which do not possess a rich repertoire of meta-communicative signals [48]. In such cases, contextual clues may be effective to avoid any misunderstanding, although what these clues are remains undetermined [58]. The use of self-handicapping, role reversal, exaggeration, and repetition also appears critical [59]. However, during particularly vigorous sessions which are consequently very risky, these subtle mechanisms may be insufficient to avoid ambiguity [55]. The short duration of rough sessions shown by resident and outgroup sifaka could be due to the lack of specific meta-communicative signals in this species [3] and, therefore, to the difficulty to maintain the playful mood.

In conclusion, our findings show that the role of play in limiting xenophobia “goes back” to the basal primate *taxon*, strepsirhines, thus revealing ancient biological roots of play in human phylogeny. In wild sifaka, play works as an ice-breaker mechanism, which enhances friendly interactions in the critical process that upgrades a stranger to a familiar individual.

## Methods

### Ethics statement

This study was approved by University of Pisa (Animal Care and Use board). Since the study was purely observational the committee waived the need for a permit. The study was conducted in the wild, with no manipulation of animals.

### Study location

We conducted this study in the gallery forest of Berenty, a 200 ha reserve on the Mandrare River in southern Madagascar (for a complete description of the study site see [60]). In particular, this research was conducted in the northern part of the forest called Ankoba (24.99°S, 46.29°E), a 40 ha secondary forest 50–60 years old, with canopy at 10e15 m (except for a few emergent acacias to more than 20 m). The site is characterized by two main climatic periods: a wet season from October to March and a dry season from April to September [60].

### Individual recognition, ingroup and outgroup animals

We observed two groups of sifaka composed of six (two adult males, one sub-adult male, two adult and one sub-adult females) and eight resident individuals (five adult males, two adult and one sub-adult females). The only infant present in one of the two groups died at the beginning of the observation period.

Non-resident adult males visited the study groups in the period around mating. Specifically, they started visiting our groups 23 days before the first mating day. We defined as “unfamiliar” nine males that were never seen with our groups in the first two months after the beginning of the observations (control period). Such males were included in the analyses as outgroup males. No outgroup female joined the group during the study period. Unfamiliar males were likely to be unrelated with most ingroup members, considering that such males mated with ingroup females, and that in *P. verreauxi* females are the phylopatric sex and group offspring is generally sired by ingroup males [61].

All resident animals were active in scent-marking, thus potentially reproductive [62]. However, lemurs undergo a transitional period in sexual maturation, indicated by a variation in the use of scent-marking (from sporadic and random to systematic) [63]. Lemurs that are not fully adult are characterized by lower marking frequencies and a smaller body size [3], which allows to be identified as subadults.

Individual recognition was based on sex and distinctive external features (scars, size, missing fur patches, fur colour, facial traits) [64]. The observational conditions (from 1 to 10 m) were excellent. In fact, animals in Berenty are well habituated to humans due to the steady presence of researchers, tourists, and local people [60].

### Observational procedures

Data were collected by I.N. and E.P. in November -December 2006 and by D.A. and a field assistant from December 2006 to February 2007 (wet season).

Before starting systematic data collection, the four observers underwent a training period during which they followed the same focal animals simultaneously and then compared the data. The training (70 h of focals) was considered as completed when the observations matched in 95% of cases [65]. At the end of the training period, Cohen's kappas (*k*) were higher than 0.70 [66]. For each behavioral category (grooming, play, and aggressive events, as explained below) we provide the kappa range (min-max) calculated for all observer dyads (six): *k*_grooming_ = 0.71–0.77; *k*_play_ = 0.74–0.81; *k*_aggression_ = 0.77–0.89. We checked again for observer reliability in December (during one day of observation), when the second dyads of observers was about to replace the first one. Also in this case, Cohen's kappas (*k*) were higher than 0.70.

After the training phase, data were collected via all-occurrences sampling methods (a total of 273 hours) [67]. The observations took place daily from dawn to dusk.

### Behavioral patterns

The behaviors recorded in this study were grooming, aggressions, and play.

Grooming, or fur-cleaning, in strepsirhines is typically performed via tooth-comb. For each grooming session we recorded groomer and groomee identity, grooming direction (who groomed who) and duration.

Aggressions involved agonistic encounters between individuals. For each aggression we recorded aggressor and aggressee identity, aggressive behavioral patterns (chasing, biting, and slapping); and submissive/frightened patterns (flee and vocalization).

For play behavior, we recorded initiator and receiver identity, play patterns (see Table 1), the duration of each play session, the behavioral pattern prior to each play session. A play session began when one partner directed any playful pattern (play invitation, PINV) towards a co-specific and ended when i) the playmates ceased their activities, ii) one of them moved away or iii) one of the two playmates was substituted by another individual. If the bout started again after a delay of 20 sec, it was counted as a new play session.

### Operational definitions

The temporary visit of outgroup males allowed us to define three different conditions: BL-IN (interactions between resident individuals with no outgroup male present), IN (interactions between resident individuals during the visit of outgroup males), OUT (interactions between resident individuals and outgroup males). Further distinction was made on the basis of the sex of interacting individuals. Male-male interactions were labeled as male-OUTmale (interactions between males of the observed groups and outgroup males), male-INmale (interactions between males of the observed groups during the visit of outgroup males), and male-BL-INmale (control variable including the interactions between males of the observed groups recorded in absence of outgroup males). Female-male interactions were labeled as female-OUTmale (interactions between females and outgroup males), female-INmale (interactions between females and ingroup males during the visit of outgroup males), and female-BL-INmale (interactions between females and ingroup males recorded in the absence of outgroup males).

On the same observation day, we calculated aggression frequencies (bouts/hour) before and after the occurrence of the first play bout. To check whether aggression rates between resident and outgroup males decreased after engaging in a play session, we compared such rates across three conditions: IN-OUTbefore-play (aggressions between resident and outgroup males before the first session of play), IN-OUTafter-play (aggressions between resident and outgroup males following the first session of play), and IN-IN (control variable including the aggressions between residents).

### Statistical analyses

The analysis was conducted at individual level. Due to the non-normal distribution of the behavioral measures (Kolmogorov-Smirnov p<.05), we used nonparametric statistics [68]. In order to avoid the bias due to the different number of individuals available for IN and OUT categories, all the frequencies (behavioral bouts over observation hours) recorded were normalized on the number of individuals belonging to the specific category, that is corrected for the number of potential partners.

The Wilcoxon signed-ranks test was used to compare the difference between: I) play frequencies between the outgroup males and the ingroup members; II) the frequencies of play invitation directed by the outgroup males towards ingroup members and vice versa; III) aggression rates before and after a play session occurred between resident and outgroup males; IV) play intensity (R_play_ and G_play_) according to the group membership of males and the median duration of play sessions within ingroup males and between ingroup and outgroup males; V) the frequencies of play sessions occurring in the presence or the absence of previous social contact (sit in contact, grooming or play) according to the group membership.

We compared, by the Friedman two-way analysis of variance, play, aggression and grooming levels across three conditions: OUTmale (interactions between males of the observed groups and outgroup males), INmale (interactions between males of the observed groups during the visit of outgroup males), and BL-INmale (control variable including the interactions between males of the observed groups recorded in absence of outgroup males). The same test was used to compare play sessions between females and outgroup males (female-OUTmale), females and ingroup males (during the visit of outgroups, female-INmale), females and ingroup males (recorded when the outgroup males were absent, female-BL-INmale). In case of significant difference between the three conditions, we applied the Dunnett's multiple comparison test (post-hoc test) to determine what pairs of conditions significantly differed [68]. We used exact two-tailed tests according to Mundry and Fischer [69].

## Supporting Information

Video S1 Rough play involving three males with a clear example of play fighting/rough and tumble (video by Ivan Norscia via Panasonic Lumix DMC FZ7 - 12× optical zoom/36–432 mm equivalent/Leica Lens)
(10.09 MB AVI)

Video S2 Play sequence between males, part of longer rough session, briefly interrupted by the arrival of a third male (video by Ivan Norscia via Panasonic Lumix DMC FZ7 - 12× optical zoom/36–432 mm equivalent/Leica Lens)
(10.19 MB AVI)

Video S3 Gentle play between resident males involving play bites (video by Daniela Antonacci via Panasonic Lumix DMC FZ7 - 12× optical zoom/36–432 mm equivalent/Leica Lens)
(9.75 MB MOV)
